# Photoprogrammable circularly polarized phosphorescence switching of chiral helical polyacetylene thin films

**DOI:** 10.1038/s41467-022-35625-3

**Published:** 2022-12-21

**Authors:** Zizhao Huang, Zhenyi He, Bingbing Ding, He Tian, Xiang Ma

**Affiliations:** Key Laboratory for Advanced Materials and Feringa Nobel Prize Scientist Joint Research Center, Frontiers Science Center for Materiobiology and Dynamic Chemistry, Institute of Fine Chemicals, School of Chemistry and Molecular Engineering, East China University of Science and Technology, Meilong Road 130, Shanghai, 200237 P. R. China

**Keywords:** Organic molecules in materials science, Self-assembly, Organic molecules in materials science

## Abstract

The developments of pure organic room-temperature phosphorescence (RTP) materials with circularly polarized luminescence (CPL) have significantly facilitated the future integration and systemization of luminescent material in fundamental science and technological applications. Here, a type of photoinduced circularly polarized RTP materials are constructed by homogeneously dispersing phosphorescent chiral helical substituted polyacetylenes into a processable poly(methyl methacrylate) (PMMA) matrix. These substituted polyacetylenes play vital roles in the propagation of CPL and present prominently optical characteristics with high absorption and luminescent dissymmetric factors up to 0.029 (g_abs_) and 0.019 (g_lum_). The oxygen consumption properties of the films under UV light irradiation endow materials with dynamic chiro-optical functionality, which can leverage of light to precisely control and manipulate the circularly polarized RTP properties with the remarkable advantages of being contactless, wireless and fatigue-resistant. Significantly, the distinct materials with dynamic properties can be used as anti-counterfeiting materials involving photoprogrammability.

## Abstract (web summary)

‘Pure organic room-temperature phosphorescence (RTP) materials with circularly polarized luminescence (CPL) gained increasing interest. Here, the authors propose a photoinduced circularly polarized RTP material by homogeneously dispersing phosphorescent chiral polyacetylenes into a processable polymer matrix.

## Introduction

Purely organic emitting materials with room-temperature phosphorescence (RTP), featuring large Stokes shifts, long-lived emission and favorable processability, have been a thriving topic in bioimaging^1,2^, sensors^3–5^, anti-counterfeiting materials^6–8^ and so forth^9–11^. Amongst the many possibilities in molecular design for RTP materials production, systematical investigation of molecular functions and material properties anchoring on chirality manipulate gradually become scientifically frontier research^12–15^. To date, exponential attention have been paid to the design and fabrication of organic optoelectronic materials with circularly polarized luminescence (CPL) functionality^16–22^, and several successful examples of circularly polarized room-temperature phosphorescent materials were designed by confining the motions of chiral aromatic molecules in the rigid crystals or polymers^23–28^.

Besides, light stimulation is particularly significant in the application of molecular switches, imaging devices, especially for the capability of contact-free remote manipulation of materials properties and inherent spatial and temporal control^29^. This sparked the inspiration to explore the possibility of constructing circularly polarized RTP materials with responsiveness to internal factors or external environment stimuli. If stimulus-responsive circularly polarized RTP materials can be exploited, the change in chiro-optical property under external stimulus could be employed as another visual monitoring parameter in addition to luminescence lifetime and color in phosphorescence, which will necessarily contribute to their practical application.

More recently, our group constructed independently circularly polarized RTP emission in an amorphous state by attaching axial chiral phosphors into polyacrylamide chains^30^. However, while the amorphous polymers with robust hydrogen bonding networks endowed the materials with high RTP quantum yield, they also limited its stimulus-response and sensitivity. Hence, we consider the modification of the rigidification in the polymer network to precisely control the photoresponsiveness. Accordingly, we develop chiral polymers comprising single-handed helical construction conveniently by taking advantage of copolymerization of chiral phNA and phosphor BrNpA. By intentionally introducing these chiral helical polymers into the polymethyl methacrylate (PMMA) matrix, the flexible films with potential circularly polarized phosphorescence emission are obtained from molding implantation. The unambiguously helical construction plays an essential role in the propagation of circularly polarized light and exhibits room-temperature phosphorescence with remarkable handedness. Besides, the oxygen consumption characteristics of PMMA under UV light irradiation endow materials with dynamic chiro-optical functionality that facilitate the control and modulation of the circularly polarized RTP properties non-invasively by light. Such photoresponsive chiral materials are considered prominent candidates for the utilization in stimulus-controllable chiroptical devices with high-optical efficiency and stabilized optical properties.

## Results

### The formation of chiral thin films

**Fig. 1:**
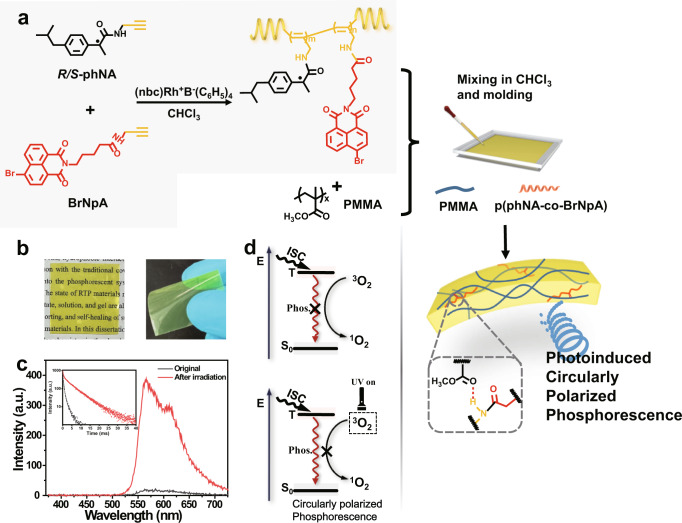
Formation of the helical films. **a** Schematic illustration of the structure and preparation of the p(phNA-co-BrNpA)-PMMA film. A flexible p(phNA-co-BrNpA) copolymer with amide group interacted with PMMA to form hydrogen bonding crosslinked network. **b** Photographs of the PMMA composite film. The films were flexible and could be bent. **c** Gated emission spectra of p(phNA_7_BrNpA_3_)-PMMA. Inset shows the phosphorescence decay curves (λ_ex_ = 365 nm, delay time = 0.1 ms). **d** Energy diagram and performance characterization of oxygen consumption in film.

As illustratively shown in Fig. 1a, the chiral polymer was constructed from chiral 4-isobutylphenyl-N-propanamide derivative (phNA) and the RTP chromophore 4-bromo-1,8-naphthalimide derivative (BrNpA) directly by the copolymerization in the presence of rhodium-based catalyst. The obtained chiral p(phNA-co-BrNpA) copolymer was analyzed by FT-IR spectroscopy and gel permeation chromatography (GPC) (Supplementary Fig. 2 and Supplementary Table 1). The successful polymerization of phNA with BrNpA can be demonstrated by the disappearance of vibrational absorption peak at about 2114 cm^−1^ from FT-IR spectra, which belongs to the symbolic absorption peak of C ≡ C group. The GPC measurement of the obtained copolymers with different phNA/BrNpA molar ratios were listed in Supplementary Table 1, which the results of moderate number-average molecular weight (Mn) and narrow molecular weight distribution (Mw/Mn) further convincingly confirmed the successful copolymerization. Besides, the actual copolymer composition was calculated by ^1^H NMR spectra. For an example shown in Supplementary Fig. 3, p(phNA_7_BrNpA_3_) was calculated to be 7:3.64, which corresponded to the theoretical value (7:3 in mol). All of the above results indicate that the required copolymers were obtained efficiently.

However, the weakly rigid environment construct by chiral polyacetylenes were difficult to inhibit both the nonradiative deactivation of the excited triplet state and oxygen quenching by blocking the oxygen diffusion into the rigid matrix well enough, only weak room-temperature phosphorescent emission was obtained (Supplementary Fig. 6). To furnish a relatively rigid environment for the chiral copolymer, the flexible p(phNA-co-BrNpA)-PMMA film with intermolecular hydrogen bonding was subsequently prepared accordingly through dissolving p(phNA-co-BrNpA) into polymethyl methacrylate (PMMA) used as a matrix. After the establishment of the ideal doping ratio of 2% (Supplementary Fig. 7), the composites films with thickness of around 225 μm were then prepared using facile film casting approach (Supplementary Fig. 5), which exhibited favorable flexibility and was easily tailored to the desired shape (Fig. 1b). The distinct photoinduced RTP emission could be discovered from the p(phNA_7_BrNpA_3_)-PMMA film, from practically non-RTP emission to the intense emission with a lifetime of 4.53 ms under continuous UV irradiation, while fluorescence emission was unaffected by the UV irradiation, indicating the important role of polymer matrix in phosphorescence effect. (Fig. 1c, Supplementary Fig. 8). For this phenomenon, the dominant factor may be the consumption of oxygen in the PMMA matrix under continuous UV irradiation, since oxygen molecules present an unusual triplet ground state, which can interact with triplet excitons and quench them (Fig. 1d).

### Photoinduced RTP behaviour

**Fig. 2:**
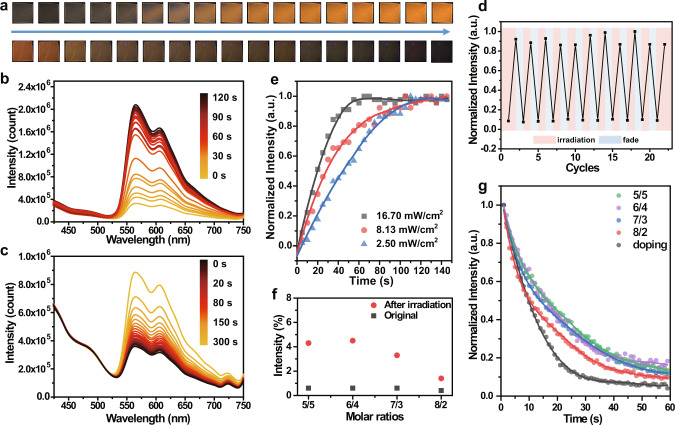
Photoinduced RTP behavior. **a** Photographic image series of p(phNA_7_BrNpA_3_)-PMMA under continuous UV light irradiation (up) and after ceasing irradiation (bottom). The photoluminescence spectra of p(phNA_7_BrNpA_3_)-PMMA under **b** sustaining UV light irradiation and **c** after ceasing irradiation (λ_ex_ = 365 nm). **d** The phosphorescence intensity at 586 nm of p(phNA_7_BrNpA_3_)-PMMA upon alternating UV light irradiation for 2 min and ceasing UV irradiation for 5 min. **e** Time-dependent normalized phosphorescence intensity of p(phNA_7_BrNpA_3_)-PMMA under UV irradiation with different power densities. **f** Quantum yield changes of p(phNA-co-BrNpA)-PMMA films with different copolymerization molar ratios before and after UV irradiation. **g** Time-dependent normalized phosphorescence quantum yield of p(phNA_7_BrNpA_3_)-PMMA with different copolymerization molar ratios after ceasing irradiation.

Expectantly, the photophysical properties of the p(phNA-co-BrNpA)-PMMA film were systematically investigated. 5/5, 6/4, 7/3 and 8/2 represented the molar ratio of phNA/BrNpA in the p(phNA-co-BrNpA)-PMMA. p(*R*-phNA_7_BrNpA_3_)-PMMA was picked out as the representative for the following elaboration. As shown in the photoluminescence spectra and the corresponding photographs, the RTP emission located at 586 nm of this film was negligible at initial state, while significantly enhanced upon continuous UV irradiation at ambient conditions. After the films were ceased UV irradiation and exposed to atmospheric conditions, triplet oxygen could permeate into the film and cause phosphorescence quenching (Fig. 2a–c and Supplementary Movie 1). Other films with different phNA/BrNpA copolymerization molar ratios exhibited approximately the same phenomenon (Supplementary Fig. 10). We next verified the stability of the observed phosphorescence photoswitching performance, as shown in Fig. 2d, the remarkably stable phosphorescent performance was demonstrated by the turning-on and -off repeated cycles of UV irradiation, which has possibilities for extensive and advanced applications in many fields. Considering the diversity of application scenarios, we quantitative investigated the influence of photoinduced RTP behavior in different power UV light. Impressively, the more easily activated RTP can be observed in higher power density of the UV lamp, which means that a greater degree of phosphorescence emission enhancement could be achieved through UV light induction in a short period of time (Fig. 2e).

To investigate the relationship between copolymerization molar ratios of the copolymer and the photoinduced RTP properties, we characterized the absolute quantum yields of the films, indicating that the quantum yields intensity increase and then decline with the amplification of the molar ratios of phNA/BrNpA in copolymer (Fig. 2f). This could be explained by the aggregation of phosphor at low phNA/BrNpA molar ratios leading to luminescence quenching, while at increasing phNA/BrNpA molar ratios, the UV-vis absorption increases leading to enhanced self-absorption (Supplementary Fig. 9). We next investigated the phosphorescent retention time of the p(phNA-co-BrNpA)-PMMA with different phNA/BrNpA ratio via monitoring fade-away process of the real-time RTP quantum yield after ceasing UV irradiation. As shown in Fig. 2g, the thin films with higher phNA/BrNpA ratios evidently presented a shorter retention time. We therefore investigated the glass transition temperature (*T*_g_) of the film by differential scanning calorimetry (DSC), and discovered the corresponding *T*_g_ values was associated with the molar ratio of phNA (Supplementary Fig. 11). This was explained by the site-resistant isobutyl group in phNA furnishes relatively steric hindrance, contributed to a steric vacancy in the physical entanglement between the polymer chains, which affects the denseness of the polymer chains and thus diminishes the rigidification effect on the phosphorescent moieties. This steric hindrance may also enhance the possibility of oxygen diffusion and permeability. Contrastingly, shorter retention time were observed for PMMA films prepared by direct doping p(phNA) with BrNpA.

### Photoinduced chiro-optical characteristic

**Fig. 3:**
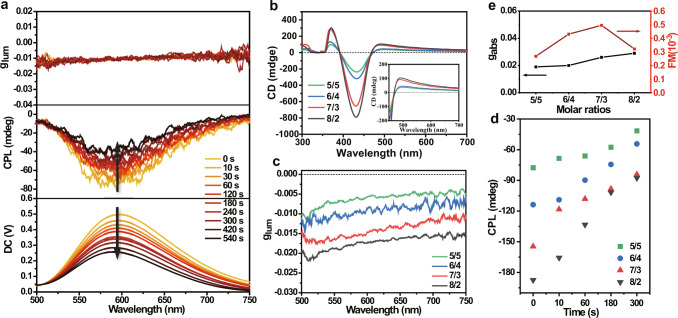
The chiro-optical properties. **a** The CPL of p(*R-*phNA_7_BrNpA_3_)-PMMA film during fade-away process (λ_ex_ = 365 nm). **b** The CD spectra of p(*R*-phNA-co-BrNpA)-PMMA film with different copolymerization molar ratios. **c** The g_lum_ spectra of p(*R*-phNA-co-BrNpA)-PMMA film with different copolymerization molar ratios. **d** Time-dependent CPL intensity (at 583 nm) of p(*R*-phNA-co-BrNpA)-PMMA with different copolymerization molar ratios after ceasing UV irradiation (according to Supplementary Fig. 19). **e** The g_abs_ and FM value of p(*R*-phNA-co-BrNpA)-PMMA film with different copolymerization molar ratios.

After confirming the copolymerization and photoinduced RTP performance of the polymers films, the circular dichroism (CD) and circularly polarized luminescence (CPL) were employed to further analyze their helical conformation. The p(*R/S*-phNA-co-BrNpA) presented intense mirror-CD signals near 425 nm in both THF and PMMA films, which demonstrated the preferred-handed helical structures of the backbones of polyacetylene (Fig. 3b, Supplementary Figs. 12 and 13). Furthermore, the intensity of CD signals enhanced significantly with the increment of *R/S*-phNA ratio in the copolymer, which was attributed to the increase in the proportion of chiral monomers during the polymerization process to improve the optical activity, and further demonstrated by the g_abs_ values (Fig. 3e). Apparently, the polymers films lacking chiral monomers phNA cannot form a predominant singlehanded helix under suitable circumstances (Supplementary Fig. 15).

CPL reflects the excited state properties of chiral materials. However, it should be mentioned that chirality and luminescence are not sufficient conditions for CPL, and it is frequent for chiral luminescent materials not to possess CPL properties, mainly due to the complexity of the chiral transfer process^31,32^. Excitingly, the CPL intensity of p(*R/S*-phNA-co-BrNpA)-PMMA film dramatically enhanced with the UV irradiation, and would return to the initial state after 5-6 min of standing under natural conditions, showing the reversible photoresponsive CPL effect, which exhibited a similar tendency with photoluminescence spectra (Fig. 3a, Supplementary Figs. 16 and 17). Ascribing to the changeless of polymer composition and conformation during the phosphorescence switching process, the dissymmetric factor g_lum_, quantified the extent of chiral dissymmetry in luminescence^33^, does not alter during this process. Besides, the increasement of phNA/BrNpA copolymerization molar ratios can improve the optical activity and thus the g_lum_, and the maximum g_lum_ can be obtained at 1.86 × 10^−2^ (Fig. 3c). Correspondingly, the polymer-PMMA films lacking chiral monomers phNA or phosphor BrNpA cannot exhibit CPL properties (Supplementary Fig. 18). As influenced by the excitation light source of the CPL spectra instrument, we shorten the spectral scanning wavelength range and sped up the scanning speed (from 100 nm/min to 500 nm/min) to investigate the CPL decay rate of different phNA/BrNpA copolymerization molar ratios (Supplementary Fig. 19). As shown in Fig. 3d, the decay law of CPL spectrum was the same as the photoluminescence spectra. Hence, the circularly polarized phosphorescent retention time can be manipulated by controlling the composition of the films. A large g_lum_ indicates well-polarized purity of the emission, while the quantum yield (PLQY) is also an essential parameter to evaluate comprehensive performance of CPL-active materials. Hence, we can propose the multiplication of the dissymmetry factor and the PLQY as a figure of merit (${{{{{\rm{FM}}}}}}={{{{{\rm{g}}}}}}{{{{{\rm{lum}}}}}}\times {{{{{\rm{PLQY}}}}}}$) for evaluating the CPL property^34^. As shown in Fig. 3e, the highest FM value of 4.9 × 10^−4^ exhibited the potential of chiral helical substituted polyacetylene in high-efficiency circularly polarized RTP materials, although there is still a long way to reach high FM values.

### The mechanism of photoinduced circularly polarized RTP

**Fig. 4:**
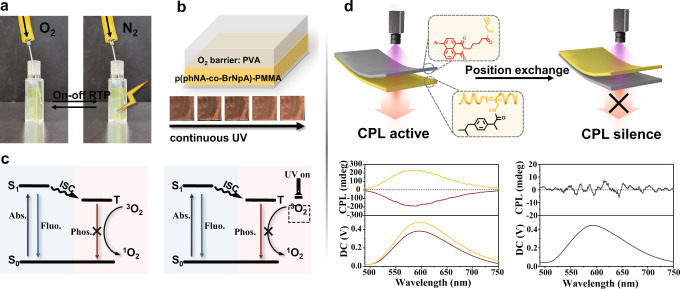
The mechanism of photoinduced circularly polarized RTP. **a** Nitrogen and oxygen were alternately filled into the quartz tube. p(*R-*phNA_7_BrNpA_3_)-PMMA exhibited RTP “turn-on” and “off” under 365 nm UV irradiation. **b** Schematic diagram and photograph of p(phNA-co-BrNpA)-PMMA coated with a layer of PVA. **c** The diagram of the internal mechanism of oxygen consumption under UV irradiation. **d** Schematic diagram of different CPL test methods (up) and the corresponding CPL spectra (bottom). The chiral helical polymers can serve as a “filter” to generate CPL.

The underlying origination of these unique photoinduced RTP effect and distinct CPL properties were explored. The phosphor BrNpA in the ground state (S_0_) absorbed the photons was excited to S_1_ state under UV irradiation, and then to the T_1_ through ISC transition. However, the highly reactive nonradiative energy transfer from the triplet state of the phosphor to triplet oxygen could lead to the quenching of phosphorescence^35–38^. After a sustaining UV irradiation over a period of time to consume the dissolved oxygen in PMMA film, then the triplet excitons could decay as phosphorescence rather than being quenched by oxygen molecules, thus producing the photoinduced RTP characteristic (Fig. 4c). To confirm the existence of a similar mechanism, the surrounding atmospheric influence on photoinduced RTP emission was investigated in nitrogen and oxygen environment, respectively. As shown in Fig. 4a and Supplementary Fig. 22, the p(phNA_7_BrNpA_3_)-PMMA film was placed in cuvette with a needle which can inject nitrogen or oxygen into the system. The film exhibited non-RTP emission even after continuous UV irradiation when the tube was kept oxygen-filled. Contrastively, the film could give a promptly response RTP to UV irradiation after replenished the tube with nitrogen. In addition, it is generally recognized that polyvinyl alcohol (PVA) possesses abundant hydrogen bonding network that prevents the phosphor from oxygen^39,40^. Therefore, we coated a layer of PVA on the surface of the material, and as can be seen from Fig. 4b, the material coated with PVA that can isolate oxygen did not exhibit photoactivatied phenomenon. Hence, it is certified that the existence of oxygen plays a significant role in the appearance of dynamic RTP behavior.

Recently, it was demonstrated that chiral helical substituted polyacetylenes can function as handedness-selective fluorescence filters to convert unpolarized fluorescence into circularly polarized luminescence by the fluorescence-selective absorption mechanism when the CD spectrum overlaps with the luminescence spectrum^41–43^. Therefore, to investigate whether the CPL signal of the films was generated because of the handed-selective fluorescence absorption of chiral helical polymers, we constructed the racemic phosphor BrNpA and nonfluorescent chiral polymer p(phNA), respectively, and then prepared them into PMMA films to implement “positioning control” CPL tests. We find that CPL could be detected when two thin films were placed in the order “light source-BrNpA films-p(phNA) film-detector”, and the intensity of CPL exhibited a positively correlated relationship with the thickness of the p(phNA) film (Supplementary Fig. 20). However, when the positions of the BrNpA film and p(phNA) film were replaced, the CPL signal totally vanished (Fig. 4d). This manifested that the relative position between the phosphor and the chiral helical polymer is significant for successful CPL construction. We next simply doped BrNpA and p(phNA) into PMMA to prepare the films and found that they also exhibited CPL properties (Supplementary Fig. 21). Experimental results indicated the existence of chiral helical polymers is essential for CPL preparation, and the CPL stemmed from the nonfluorescent chiral helical polymers “filter” affection.

### Applications of the chiral thin films

**Fig. 5:**
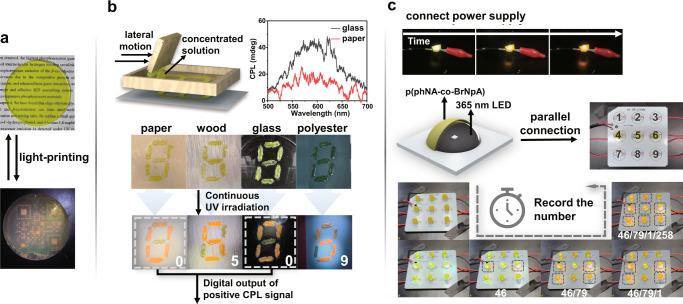
Applications of photoprogrammable circularly polarized RTP materials. **a** Photographs of the p(phNA-co-BrNpA)-PMMA with light-printing QR code pattern in front of a photomask under 365 nm light. **b** Manufacturing process of the anti-counterfeiting patterns and the CPL spectrum of the patterns (top). The pattern of “8” was generated on each material using p(phNA)-PMMA first, and the hidden pattern was printed again using p(*R/S*-phNA_7_BrNpA_3_)-PMMA. Photographs of hidden pattern taken under UV irradiation (bottom). **c** Photographs of the fabrication and properties of the smart photoelectric material. When connected to the power supply, record the number corresponding to the lighted bulb as time progresses. First is the number 46, followed by 79, 1 and 258.

These controllable luminescent properties and the flexibility and plasticity of the materials enable the construction of advanced smart photonic technologies. Hence, under the UV light irradiation, the formation of the PMMA film can perform the light-printing properties (Fig. 5a). Besides, we fabricated the self-erasable transient information storage and anti-counterfeiting in various daily materials by utilizing facile stencil printing with the p(*R/S*-phNA_7_BrNpA_3_)-PMMA concentrated solution as paint to generate the information patterns. As presented in Fig. 5b, the patterns numbers were invisible under natural light on paper, wood, glass and polyester, which was increasingly uncovered with orange emission under continuous 365 nm UV irradiation. Importantly, while the number “0/5/0/9” exhibited a similar emission color under continuous 365 nm UV irradiation, the positive CPL signal can be observed from the number “0/0”, but not from the number “5/9”. This provided anti-counterfeiting information involving CPL signal with more application scenarios. In addition, we conveniently prepared the materials into lampshade by adding p(phNA-co-BrNpA)-PMMA CHCl_3_ suspension to a stencil mask, then drying the suspension and removing the stencil mask. When connected to the power supply and the LED was turned on at 5.5 V, the lampshade placed on the 365 nm commercial LED will gradually light up with bright orange emission. Then we utilize nine small bulbs connected in parallel to form a lamp panel and artificially labeled with numbers. And using different copolymerization molar ratios of the materials to fabricate the lampshades. As shown in Fig. 5c and Supplementary Movie 2, the lampshades will have different times of being illuminated when the lamp panel was connected to the power supply. Therefore, it is possible to detect different digital patterns at different time scales, which can be potentially employed as a time-informed anti-counterfeiting technology.

## Discussion

In summary, we exploited a type of photoinduced circularly polarized RTP materials by homogeneously dispersing phosphorescent chiral helical substituted polyacetylenes into an rigid poly(methyl methacrylate) matrix. Benefiting from the chiral polyacetylenes with a predominant singlehanded helix, the flexible films exhibited prominent handedness optical properties. The oxygen consumption characteristics of the concomitant PMMA under UV light irradiation generated significant dynamic chiro-optical modulation. Based on the investigation of the structure-activity relationship, we can exploit light to precisely control and manipulate the circularly polarized RTP properties involving photoprogrammability, which enriched the functionality of RTP materials. This renders these films competitive candidates for the promising chiro-optical applications with dynamic properties and favorable processability.

## Methods

### Materials

All reagents and solvents employed were commercially available and used as received without further purification. 4-Bromo-1,8-naphthalic anhydride (Adamas-beta), which was purified by column chromatography (ethyl acetate/cyclohexane, 1/1, v/v). Solvents were purified according to standard laboratory methods. The molecular structures were confirmed using ^1^H NMR, ^13^C NMR and high-resolution ESI mass spectroscopy.

### General methods

The UV-Vis absorption spectra and PL spectra were performed on a Varian Cray 500 spectrophotometer and a Horiba Fluoromax-4 at 25 °C, respectively. Phosphorescence spectra and phosphorescence lifetimes were obtained on a Varian Cary Eclipse spectrophotometer. Quantum yields were measured by using an integrating sphere on a HAMAMATSU Quantaurus-QY C11347-11. Powder X-ray diffraction (XRD) was performed on a D/max2550VB/PC. Circular dichroism (CD) spectra were acquired using the JASCO J815 spectrophotometer. CPL spectra were acquired using the JASCO CPL-200 spectrofluoropolarimeter. GPC was performed on a Series 200. Except special instructions, the UV irradiation source used was from tunable 365 nm LED lamp with 16.7 mW/cm^2^.

### Reporting summary

Further information on research design is available in the Nature Portfolio Reporting Summary linked to this article.

## Supplementary information

Supplementary Information

Description of Additional Supplementary Files

Supplementary Movie 1

Supplementary Movie 2

## Data Availability

All relevant data that support the findings are available within this article and supplementary information and are also available from authors upon request. Source data are available. Source data are provided with this paper.

## Source data

Source Data
