# Supplementary material for: Photoprogrammable circularly polarized phosphorescence switching of chiral helical polyacetylene thin films
Source: Nat Commun. 2022 Dec 21;13:7841. doi: 10.1038/s41467-022-35625-3 (PMC9772410; doi:10.1038/s41467-022-35625-3)
Supplement: Supplementary file 1 — Supplementary Information [file 41467_2022_35625_MOESM1_ESM.pdf]

Supplementary Information

Photoprogrammable Circularly Polarized Phosphorescence Switching of Chiral Helical Polyacetylene Thin Films

Zizhao Huang[1], Zhenyi He[1], Bingbing Ding[1], He Tian[1] and Xiang Ma[1]*

[1]Key Laboratory for Advanced Materials and Feringa Nobel Prize Scientist Joint Research Center, Frontiers Science Center for Materiobiology and Dynamic Chemistry, Institute of Fine Chemicals, School of Chemistry and Molecular Engineering, East China University of Science and Technology, Meilong Road 130, Shanghai 200237, P. R. China.

Email: maxiang@ecust.edu.cn

1. Synthesis

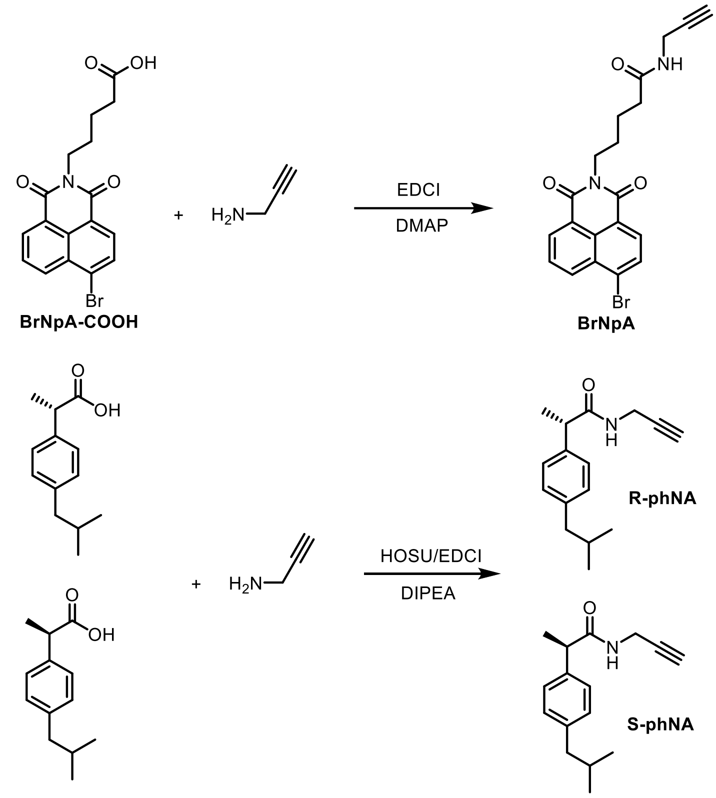

Supplementary Figure 1. Synthetic routes of monomer.
(R)/(S)-2-(4-isobutylphenyl)propanoic acid were purchased from TCI Chemicals and used without further purification.

(*R*)/(*S*)-2-(4-isobutylphenyl)-N-(prop-2-yn-1-yl)propenamide (*R/S*-phNA): (R)/(S)-2-(4-isobutylphenyl)propanoic acid (100 mg, 0.49 mmol, 1 eq), N-Hydroxy succinimide (HOSU) (67 mg, 0.582 mmol, 1.2 eq) and EDC·HCl (111.5 mg, 0.582 mmol, 1.2 eq) in DCM (100 ml). After the reaction mixture was stirred for 30 min, mono-Propargylamine (32 mg, 0.582 mmol, 1.2eq) and *N,N*-Diisopropylethylamine (DIPEA) (188 mg, 1.45 mol, 3 eq) was added to the above solution. The reaction mixture was stirred overnight at room temperature. The reaction was quenched with water, extracted with DCM, dried over $MgSO_4$, filtered, concentrated, and purified by column chromatography on silica (DCM: EA = 30:1) to give white solid 86 mg. Yield: 66.3%. ^{1}H NMR (400 MHz, Chloroform-*d*, 298 K) δ 7.19 (d, *J* = 8.2 Hz, 2H), 7.13 (d, *J* = 8.1 Hz, 2H), 5.51 (s, 1H), 4.09 – 3.88 (m, 2H), 3.55 (q, *J* = 7.2 Hz, 1H), 2.46 (d, *J* = 7.2 Hz, 2H), 2.17 (t, *J* = 2.5 Hz, 1H), 1.93 – 1.78 (m, 1H), 1.52 (d, *J* = 7.3 Hz, 3H), 0.90 (d, *J* = 6.6 Hz, 6H). ^{13}C NMR (101 MHz, Chloroform-*d*, 298 K) δ 174.04, 140.95, 138.03, 129.74, 127.41, 79.53, 71.46, 46.58, 45.02, 30.19, 29.38, 22.39, 18.43. HRMS (ESI) m/z: $[M + Na]^+$ calculated for $C_{16}H_{21}NNaO^+$, 266.1515; found, 266.1521.

5-(6-bromo-1,3-dioxo-1H-benzo[de]isoquinolin-2(3H)-yl)-N-(prop-2-yn-1-yl)pentanamide (BrNpA): A mixture of **BrNpA-COOH** (100 mg, 0.27 mmol, 1eq), mono-Propargylamine (17.8 mg, 0.32 mmol, 1.2 eq), EDC·HCl (61.3 mg, 0.32 mmol, 1.2 eq), and DMAP (39.1 mg, 0.32 mmol, 1.2 eq) in DCM (100 ml) was stirred at rt for 5 h, quenched with aqueous water, extracted with DCM, dried over $MgSO_4$, filtered, concentrated, and purified by column chromatography on silica (DCM: EA = 1:1) to give white solid 78 mg. Yield: 79.3%. ^{1}H NMR (400 MHz, Chloroform-*d,* 298 K) δ 8.66 (dd, *J* = 7.3, 1.2 Hz, 1H), 8.58 (dd, *J* = 8.5, 1.1 Hz, 1H), 8.42 (d, *J* = 7.8 Hz, 1H), 8.05 (d, *J* = 7.8 Hz, 1H), 7.86 (dd, *J* = 8.5, 7.3 Hz, 1H), 5.94 (s, 1H), 4.21 (t, *J* = 6.8 Hz, 2H), 4.05 (dd, *J* = 5.2, 2.6 Hz, 2H), 2.33 (t, *J* = 7.1 Hz, 2H), 2.20 (t, *J* = 2.6 Hz, 1H), 1.83 – 1.72 (m, 4H). ^{13}C NMR (101 MHz, Chloroform-*d,* 298 K) δ 172.36, 163.76, 138.96, 133.45, 132.19, 131.37, 131.15, 130.69, 130.46, 129.05, 128.13, 123.02, 122.14, 79.72, 71.49, 39.53, 35.71, 29.19, 27.31, 22.94. HRMS (ESI) m/z: $[M + Na]^+$ calculated for $C_{20}H_{17}BrN_2NaO_3^+$, 435.0315; found, 435.0320.

Synthesis of the Polymer p(phNA-co-BrNpA): According to the copolymerization molar ratio, the monomer R/S-phNA, BrNpA and the catalyst $(nbd)Rh^+B^-(C_6H_5)_4$ were dissolved in 2 mL of chloroform ($CHCl_3$). The polymerization was performed under the following conditions: [monomer]/[catalyst] =100, N_2 at 30 ^{0}C for 6 h. The

resulting mixture was added into methanol to precipitate polymeric materials. The crude product was washed with methanol 2–3 times to give purified polymers.

Synthesis of the corresponding PMMA Films: The copolymers (20 mg), and PMMA (1 g) were dissolved in 10-20 mL $CHCl_3$ solution. The mixture was further stirred for 3 h to remove excess $CHCl_3$, and formed a homogenous dispersed solution. The solution was transferred to a template container, placed in air for 10 min, and then dried sequentially in a 40 °C oven for another 3 h. The transparent and flexible p(phNA-co-BrNpA)-PMMA film were obtained.

2. Characterization of the structure of monomer and copolymer

(R-phNA was picked out as the representative for the following elaboration.**)**

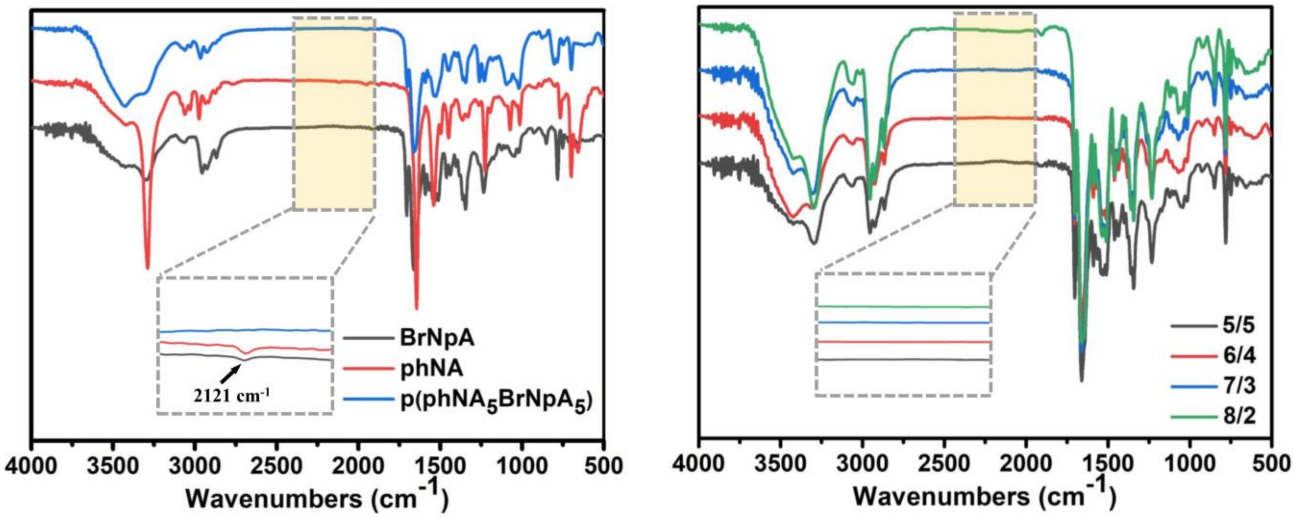

Supplementary Figure 2. The FT-IR spectra of **phNA, BrNpA** and **p(phNA-co-BrNpA)** with different copolymerization molar ratios. (KBr tablet).

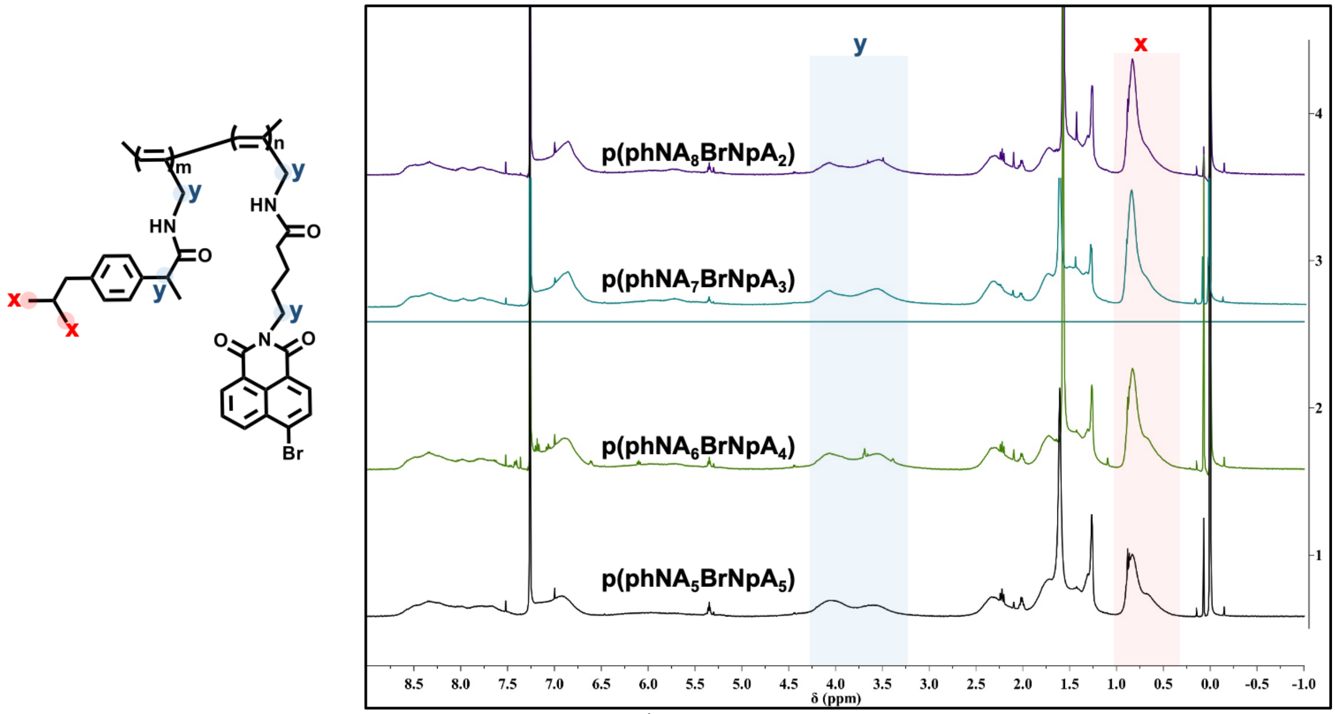

Supplementary Figure 3. The Chemical structure and ^{1}H NMR spectra of the **p(phNA-co-BrNpA)**.

Supplementary Table 1. Polymer composition and properties of the synthesized copolymers.

polymer	Composition (mol%)[a)]	Mn[b)]	Mw[b)]	D[b)]
p(phNA$_5$BrNpA$_5$)	5:4.41	29262	49724	1.699
p(phNA$_6$BrNpA$_4$)	6:3.81	20829	37538	1.802
p(phNA$_7$BrNpA$_3$)	7:3.64	25978	71627	2.757
p(phNA$_8$BrNpA$_2$)	8:2.82	21173	55391	2.616

[a)] Proportions of **phNA** and **BrNpA** in the copolymers as determined by ^{1}H NMR (Supplementary Figure 3) spectroscopy.
[b)] Determined by GPC.

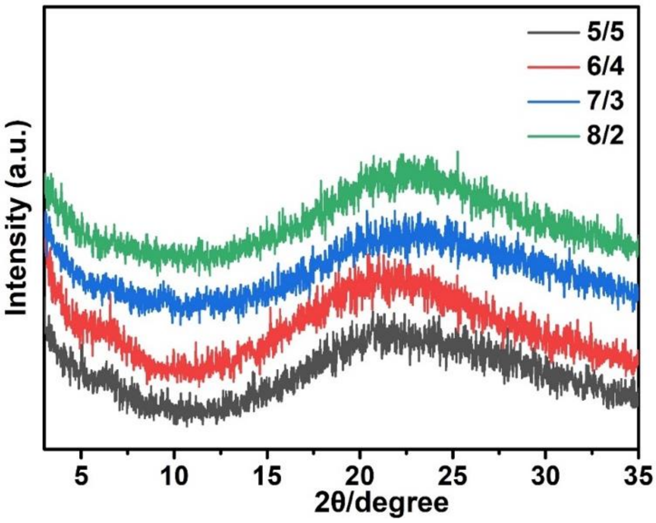

Supplementary Figure 4. The XRD patterns of **p(phNA-co-BrNpA)** with different copolymerization molar ratios.

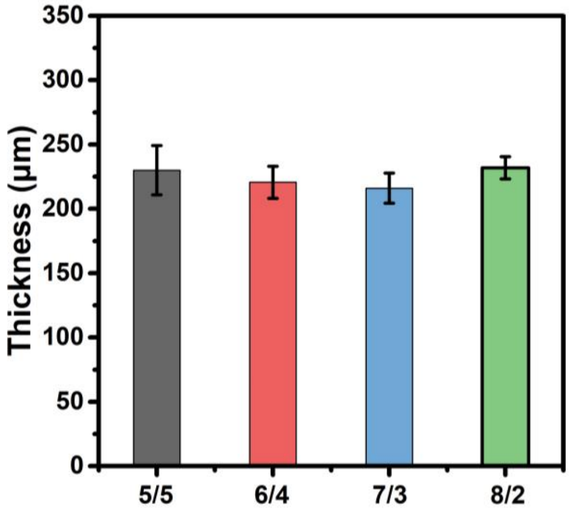

Supplementary Figure 5. The thickness of the **p(phNA-co-BrNpA)-PMMA** film. Data with error bars are expressed as mean ± s.d., n = 5 independent samples.

3. Optical properties of the materials

(***R*-phNA** was picked out as the representative for the following elaboration.)

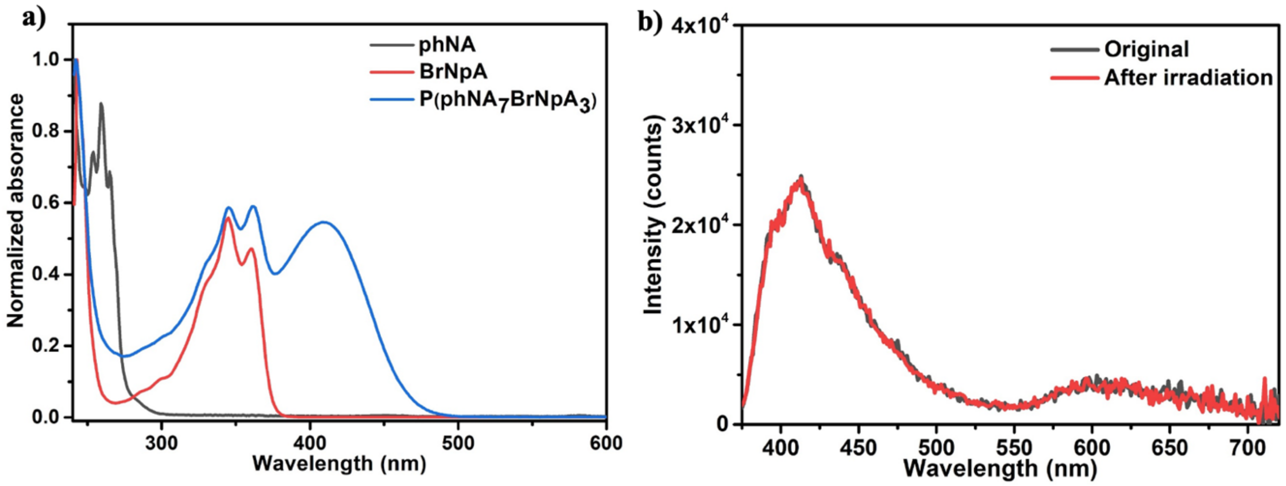

Supplementary Figure 6. a) The normalized absorption spectra of **phNA, BrNpA** and **p(phNA$_7$BrNpA$_3$)**. b) The photoluminescence spectra of **p(phNA$_7$BrNpA$_3$).**

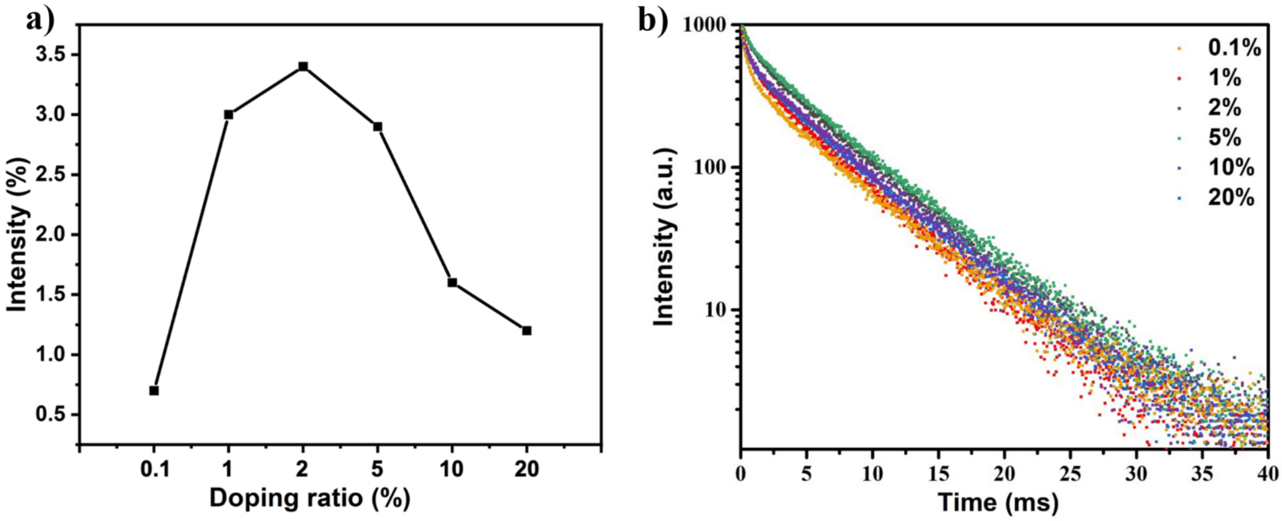

Supplementary Figure 7. a) Quantum yield and b) phosphorescence decay curves (λ_{ex}=365 nm, delay time=0.1 ms) of **p(phNA$_7$BrNpA$_3$)-PMMA** films with difference doping ratio.

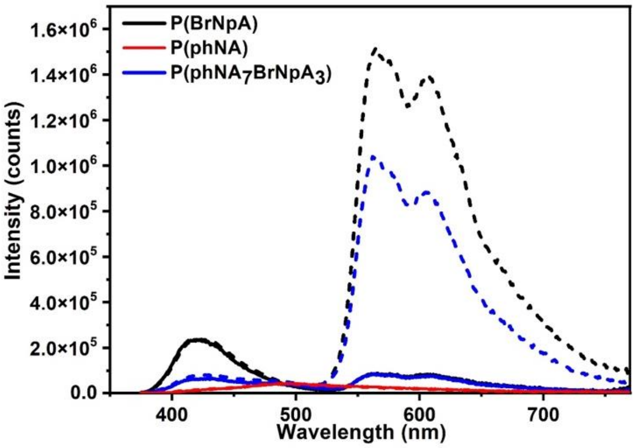

Supplementary Figure 8. The photoluminescence spectra of **p(phNA)-PMMA, BrNpA-PMMA** and **p(phNA-co-BrNpA)-PMMA** film before (solid lines) and after (broken lines) continuous UV light irradiation.

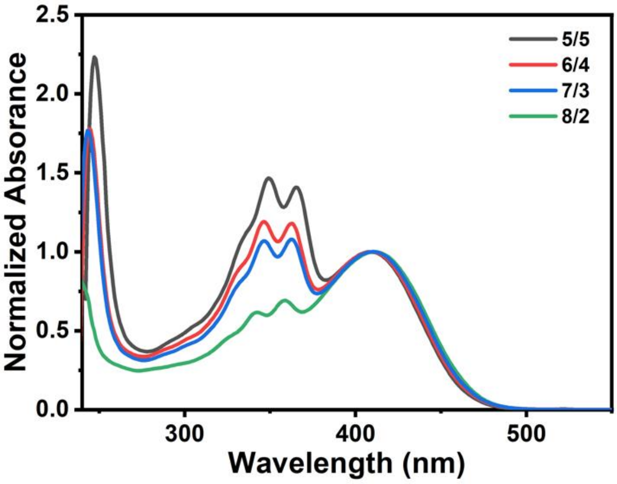

Supplementary Figure 9. The normalized absorption spectra of **p(*R*-phNA-co-BrNpA)-PMMA** film with different copolymerization molar ratios.

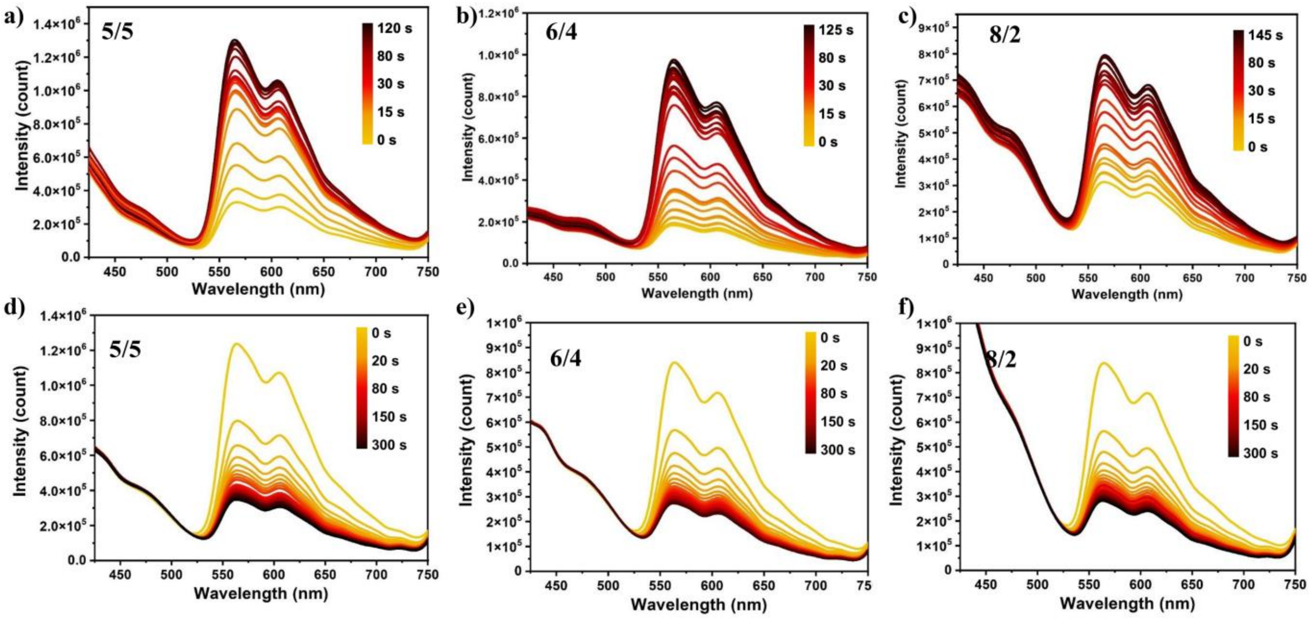

Supplementary Figure 10. The photoluminescence spectra of **p(phNA-co-BrNpA)-PMMA** under a), b), c) sustaining UV light irradiation and d), e), f) after ceasing irradiation.

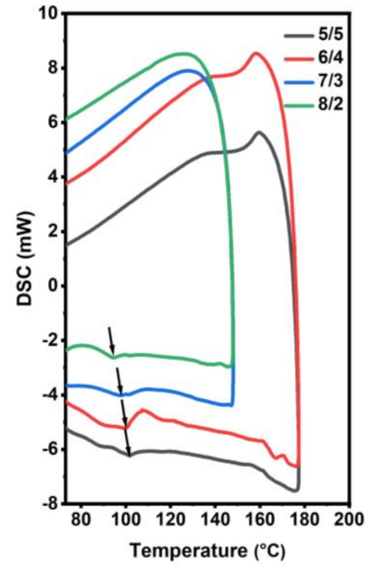

Supplementary Figure 11. DSC thermograms of **p(*R*-phNA-co-BrNpA)-PMMA** film with different copolymerization molar ratios.

4. Chiro-optical properties of the materials

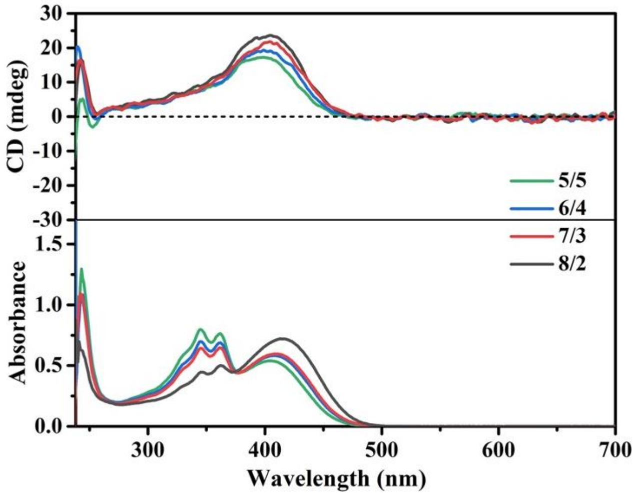

Supplementary Figure 12. The CD spectra of **p(*S*-phNA-co-BrNpA)** with different copolymerization molar ratios in THF.

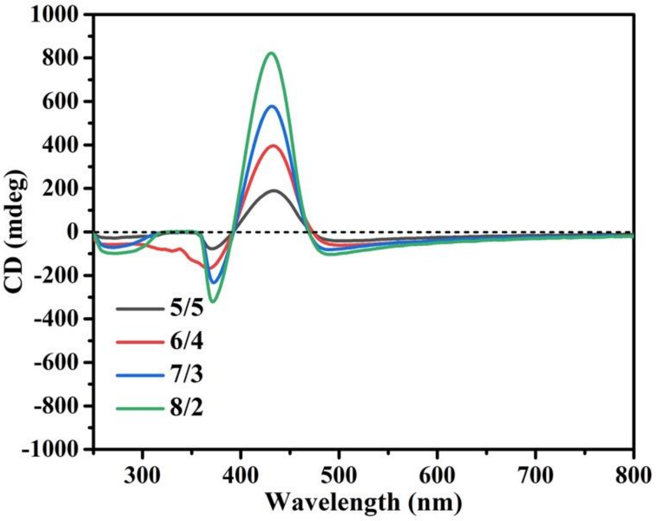

Supplementary Figure 13. The CD spectra of **p(*S*-phNA-co-BrNpA)-PMMA** with different copolymerization molar ratios.

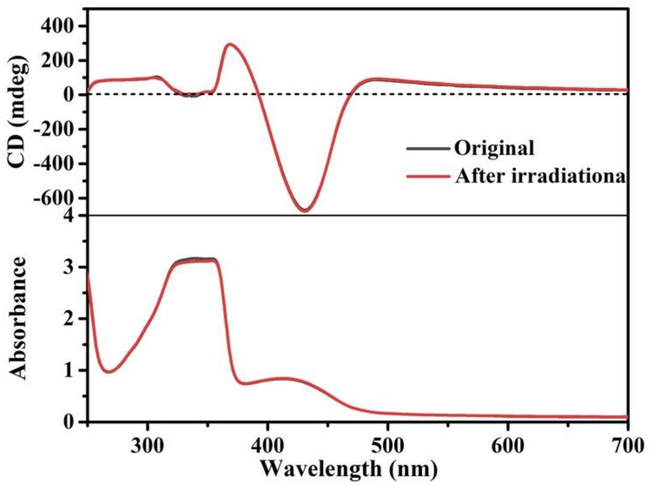

Supplementary Figure 14. The CD spectra of **p(*R*-phNA$_7$BrNpA$_3$)-PMMA** before and after continuous UV light irradiation.

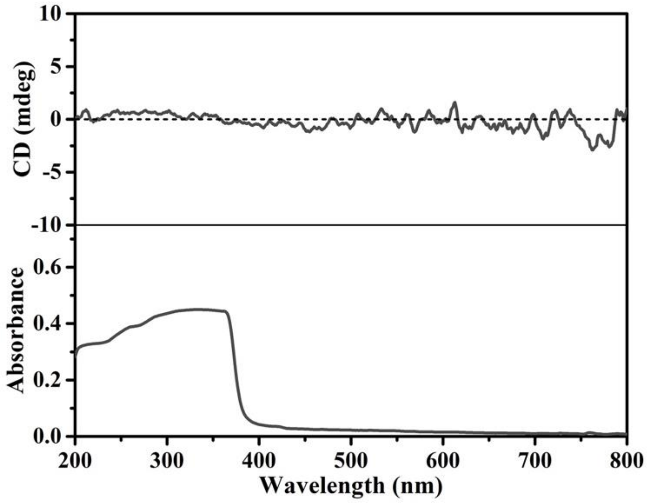

Supplementary Figure 15. The CD spectra of **p(BrNpA)-PMMA.**

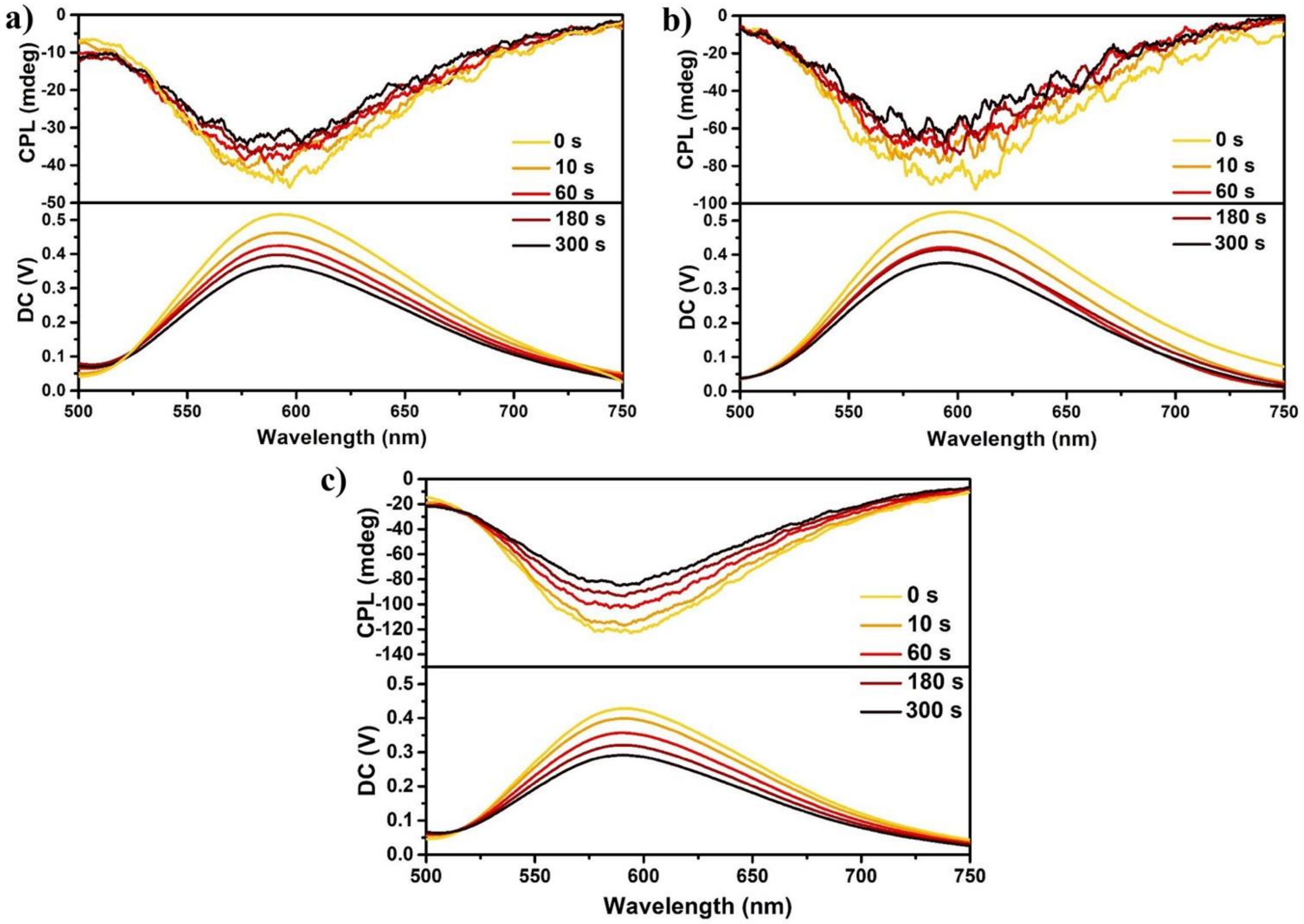

Supplementary Figure 16. The CPL spectra of **p(R-phNA-co-BrNpA)-PMMA** with a) 5/5, b) 6/4, c) 8/2 copolymerization molar ratios.

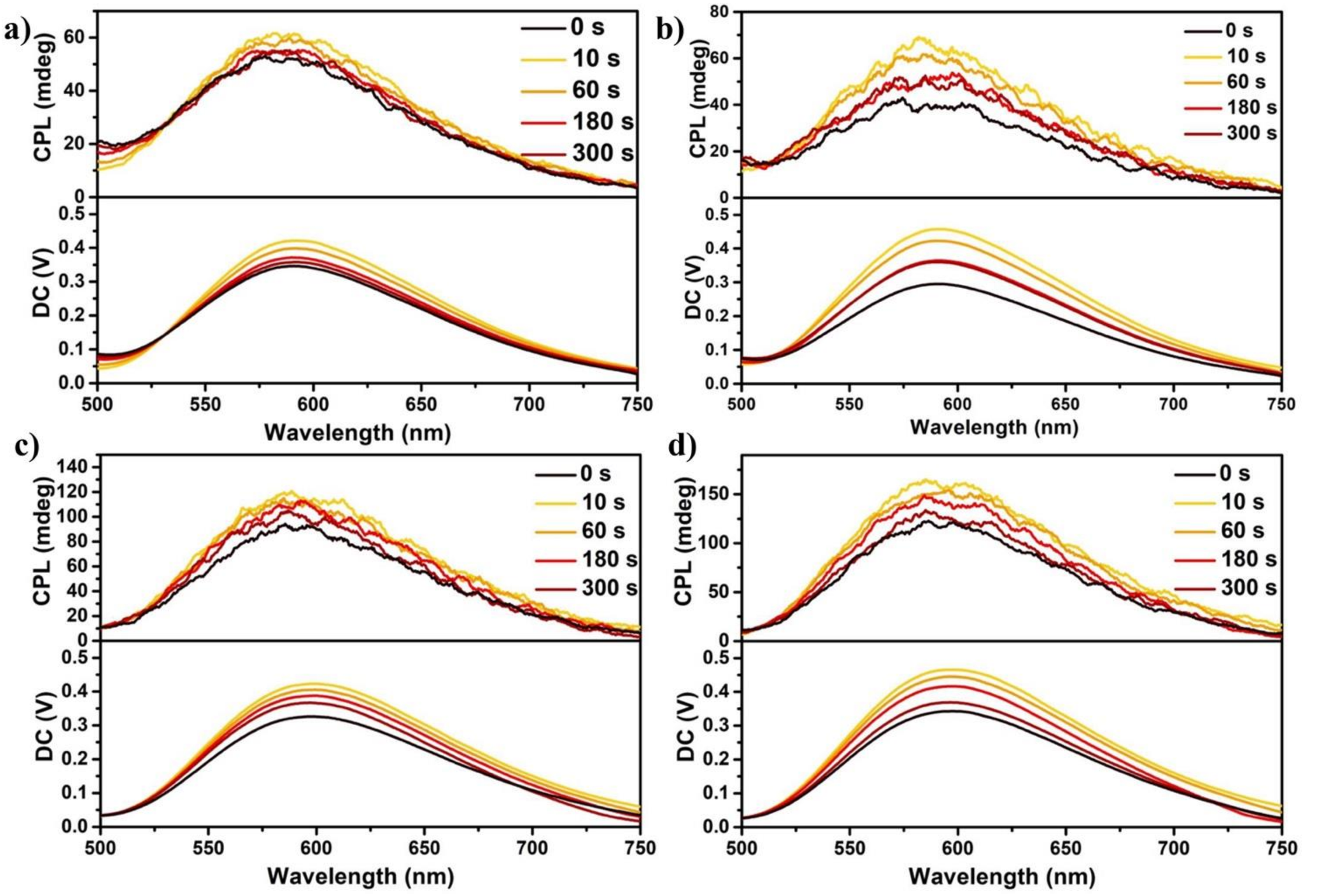

Supplementary Figure 17. The CPL spectra of **p(*S*-phNA-co-BrNpA)-PMMA** with a) 5/5, b) 6/4, c) 7/3 d) 8/2 copolymerization molar ratios.

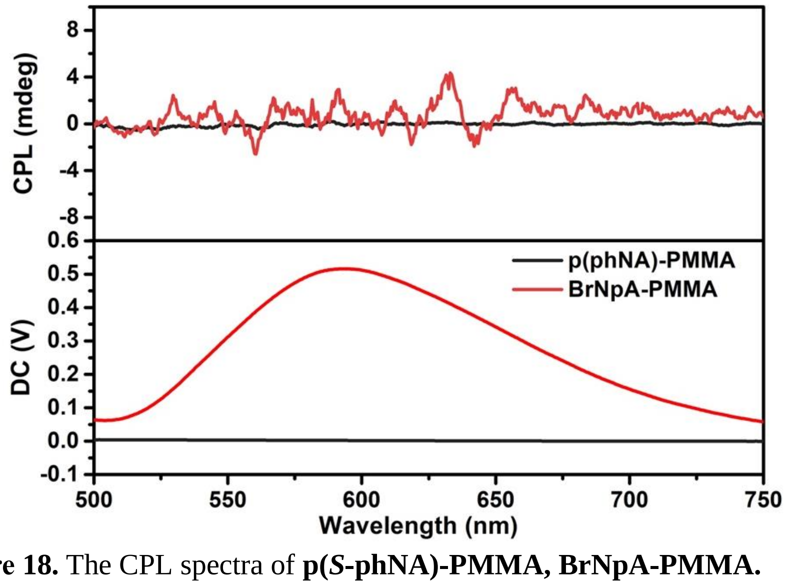

Supplementary Figure 18. The CPL spectra of **p(*S*-phNA)-PMMA, BrNpA-PMMA.**

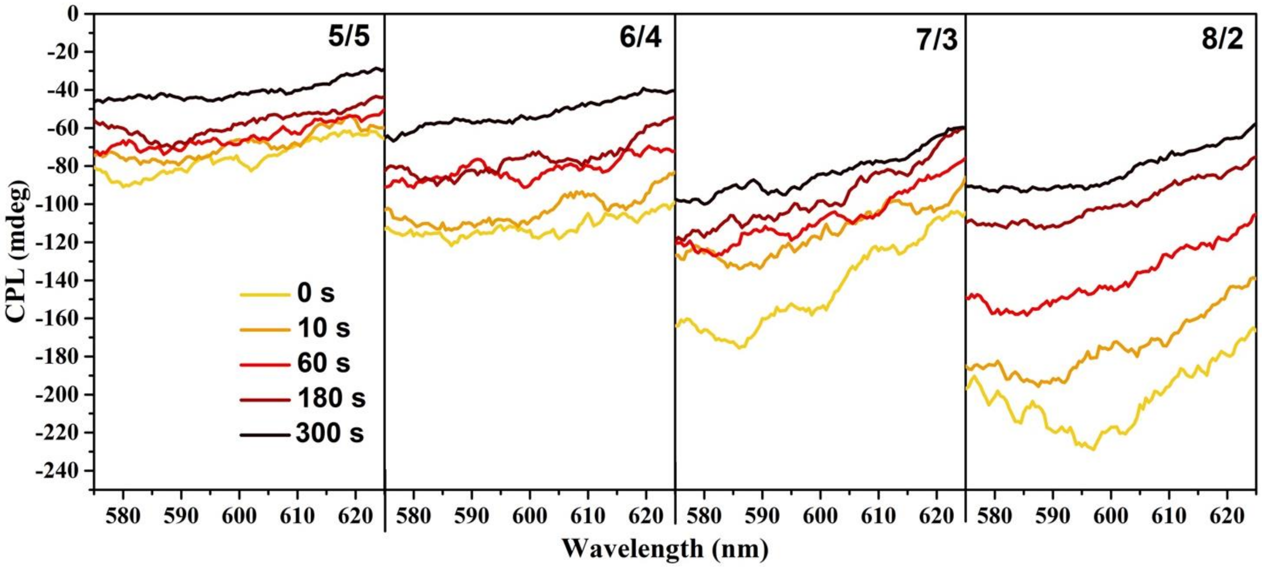

Supplementary Figure 19. The CPL spectra of **p(*R*-phNA-co-BrNpA)-PMMA** with copolymerization molar ratios under high scanning speed (500 nm/min).

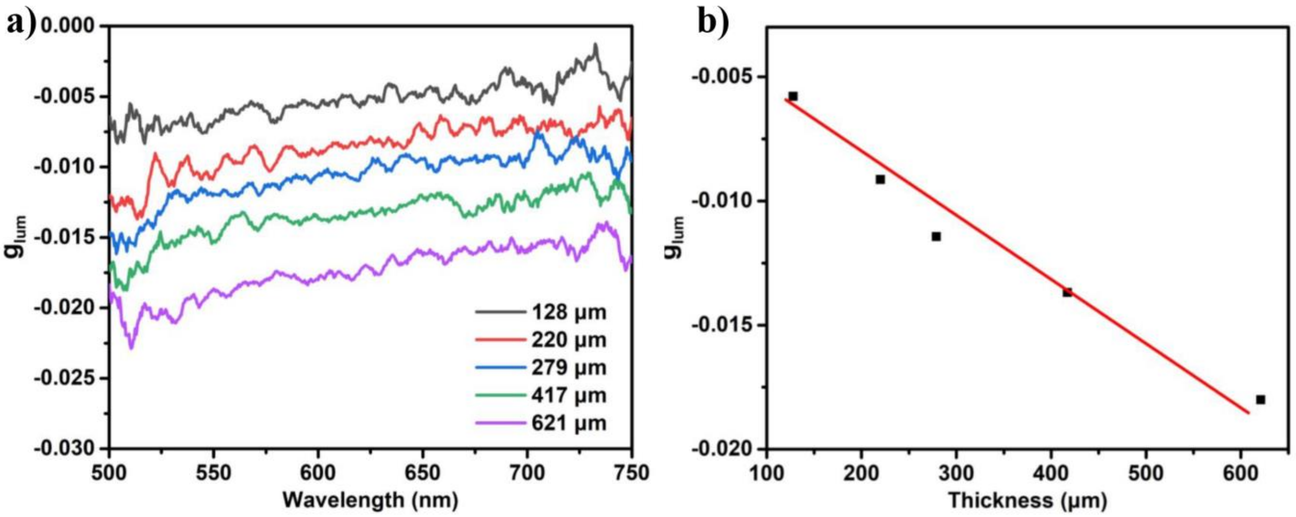

Supplementary Figure 20. (a) The g_{lum} spectra in "positioning control" CPL tests with different **p(*R*-phNA)** film thickness. (b) Corresponding plot of g_{lum} vs film thickness. The straight lines were fitted ones by Origin 2021.

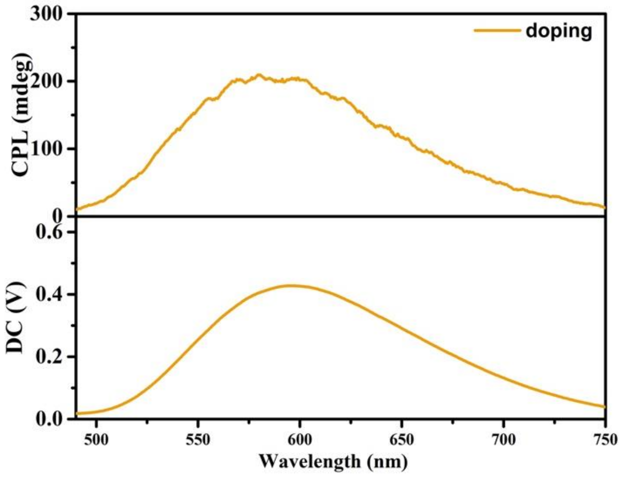

Supplementary Figure 21. The CPL spectra of **BrNpA** doping in **p(*S*-phNA)-PMMA.**

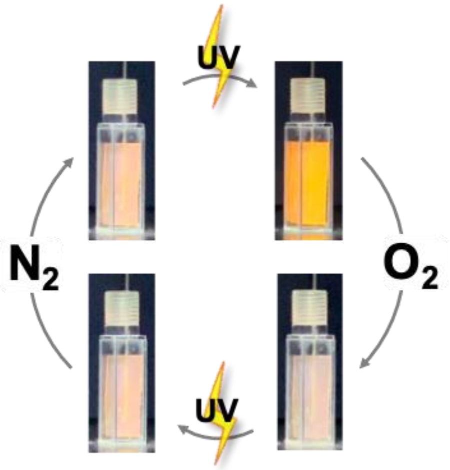

Supplementary Figure 22. The photograph of nitrogen and oxygen alternately filling the quartz tube.

5. ^{1}H NMR, ^{13}C NMR and high-resolution ESI mass spectroscopy

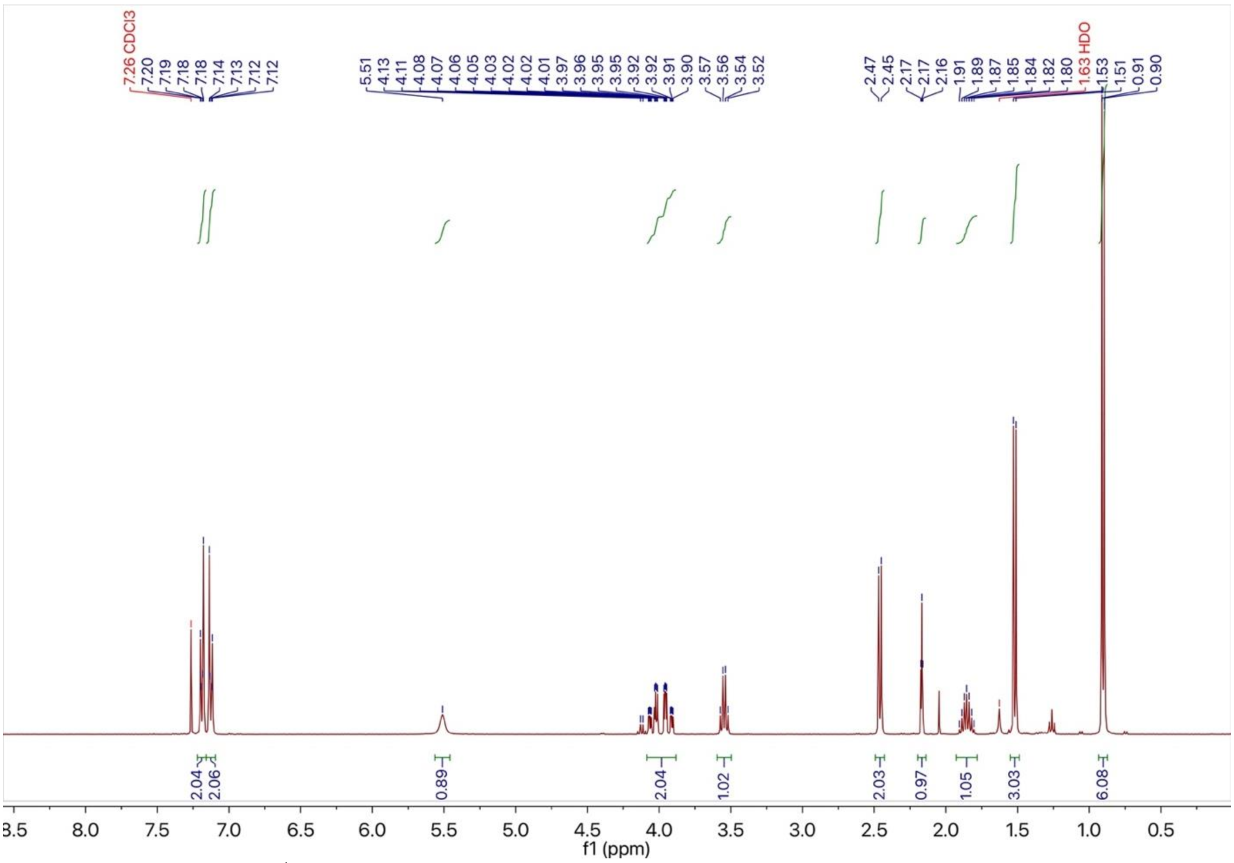

Supplementary Figure 23. ^{1}H NMR spectrum (400 MHz) of ***R*-phNA** in $CDCl_3$.

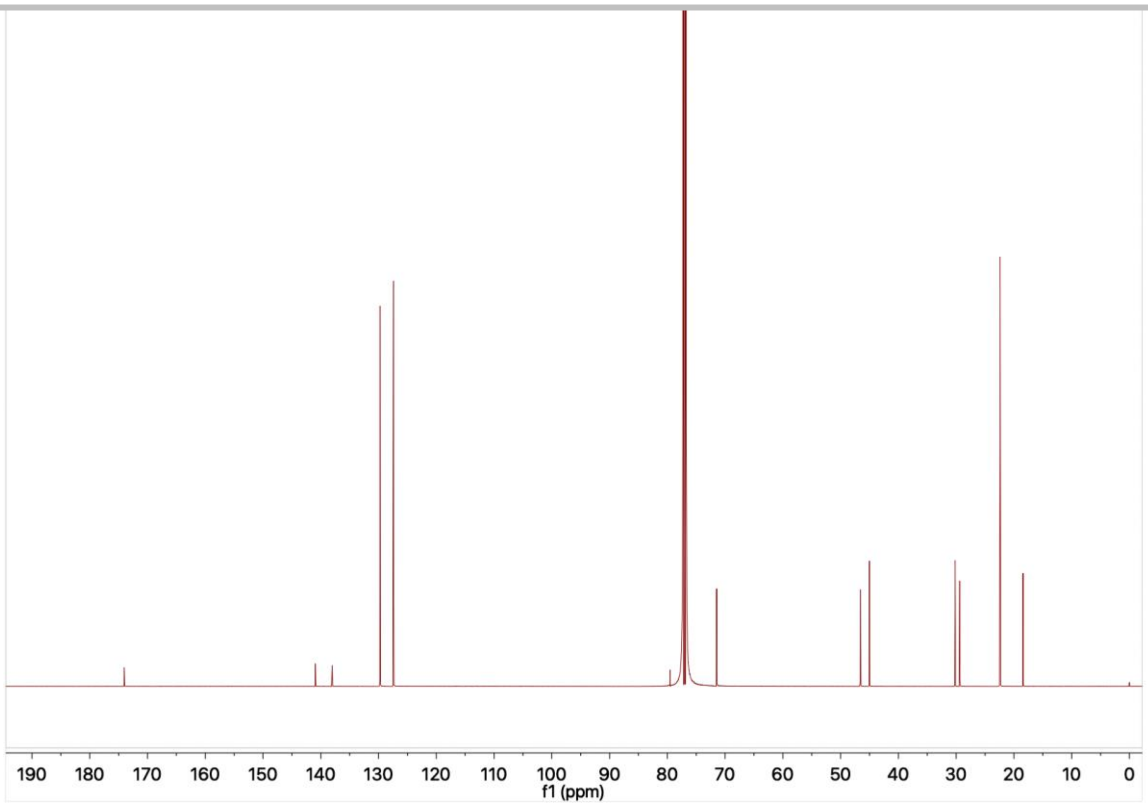

Supplementary Figure 24. ^{13}C NMR (101 MHz) spectrum of ***R*-phNA** in $CDCl_3$.

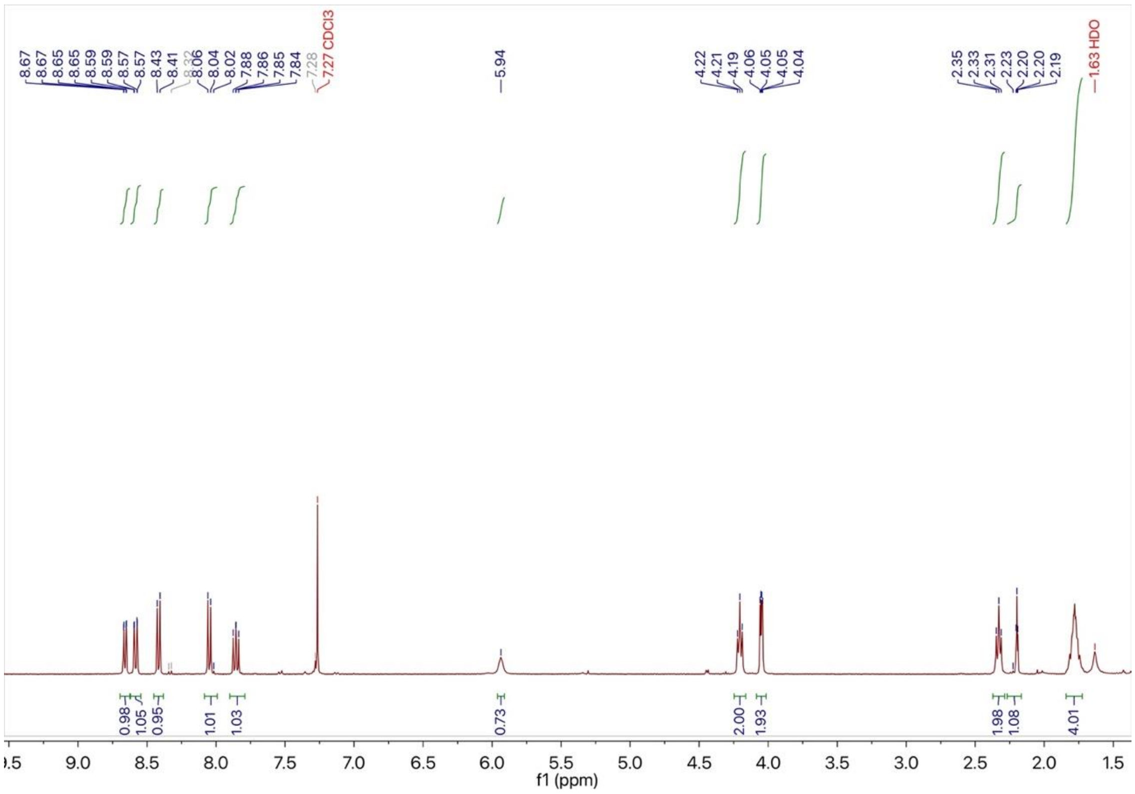

Supplementary Figure 25. ^{1}H NMR spectrum (400 MHz) of **BrNpA** in $CDCl_3$.

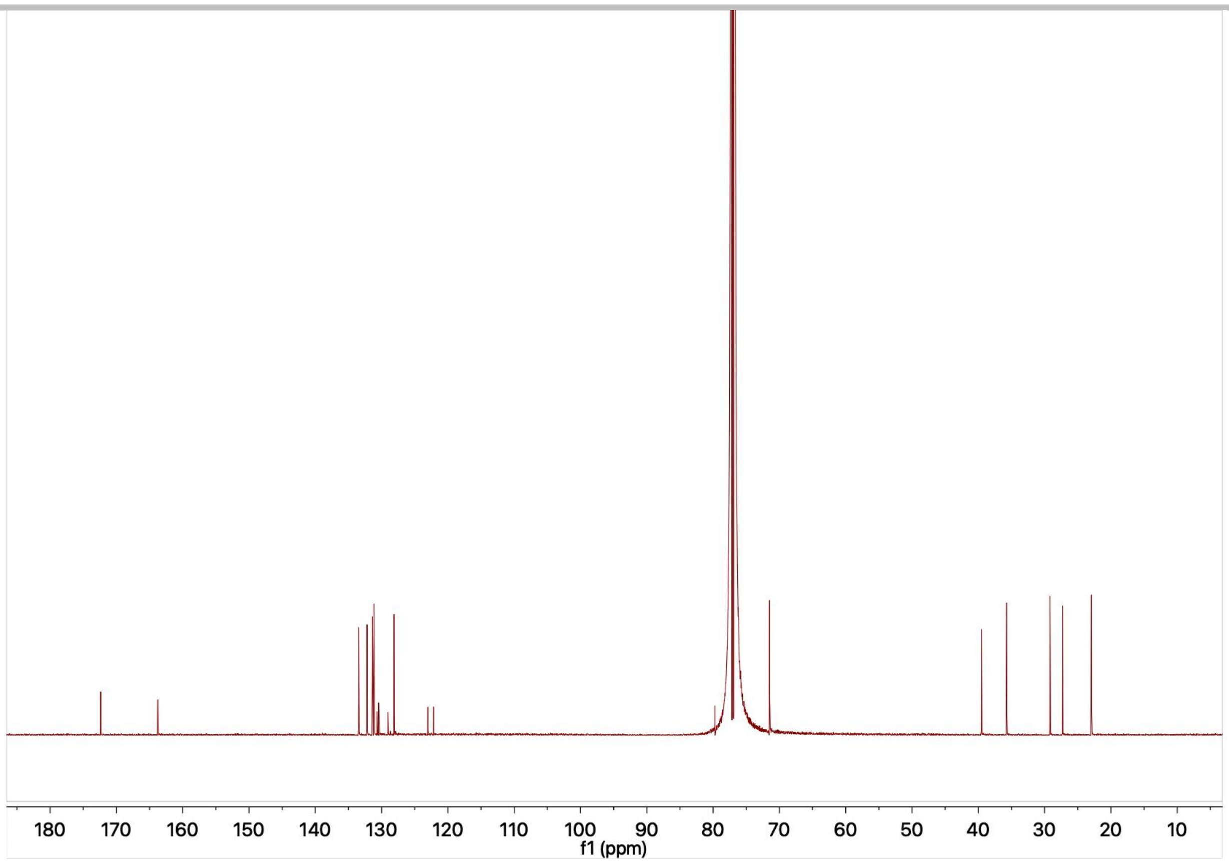

Supplementary Figure 26. ^{13}C NMR spectrum (101 MHz) of **BrNpA** in $CDCl_3$.

Elemental Composition Report **Page 1**

Single Mass Analysis
Tolerance = 15.0 PPM / DBE: min = -1.5, max = 50.0
Element prediction: Off
Number of isotope peaks used for i-FIT = 3

Monoisotopic Mass, Even Electron Ions
42 formula(e) evaluated with 1 results within limits (up to 50 closest results for each mass)
Elements Used:
C: 0-20 H: 0-17 N: 0-2 O: 0-3 Na: 0-1 Br: 0-1

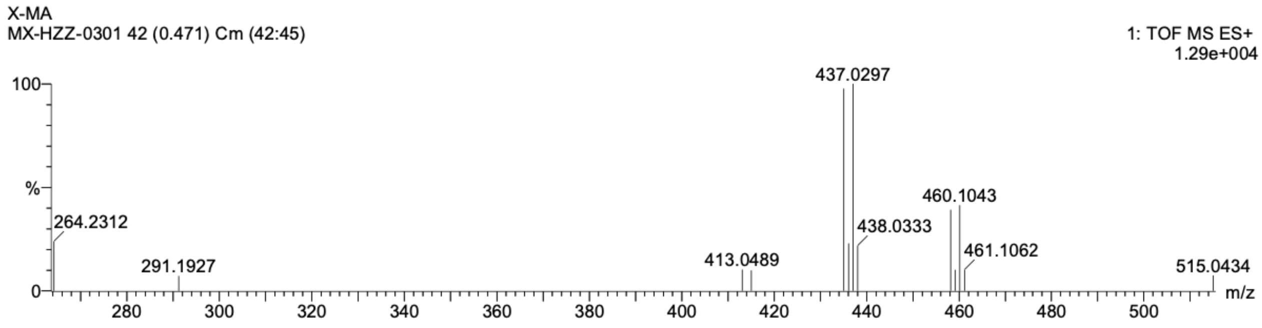

Minimum:				-1.5			
Maximum:		5.0	15.0	50.0			
Mass	Calc. Mass	mDa	PPM	DBE	i-FIT	i-FIT (Norm)	Formula
435.0302	435.0320	-1.8	-4.1	12.5	9.0	0.0	C20 H17 N2 O3 Na Br

Supplementary Figure 27. HRMS (ESI) spectrum of **BrNpA**.

Elemental Composition Report **Page 1**

Single Mass Analysis
Tolerance = 15.0 PPM / DBE: min = -1.5, max = 50.0
Element prediction: Off
Number of isotope peaks used for i-FIT = 3

Monoisotopic Mass, Even Electron Ions
5 formula(e) evaluated with 1 results within limits (up to 50 closest results for each mass)
Elements Used:
C: 0-16 H: 0-21 N: 0-1 O: 0-1 Na: 0-1
X-MA
MX-HZZ-0302 67 (0.756) Cm (67:70) 1: TOF MS ES+ 3.25e+004

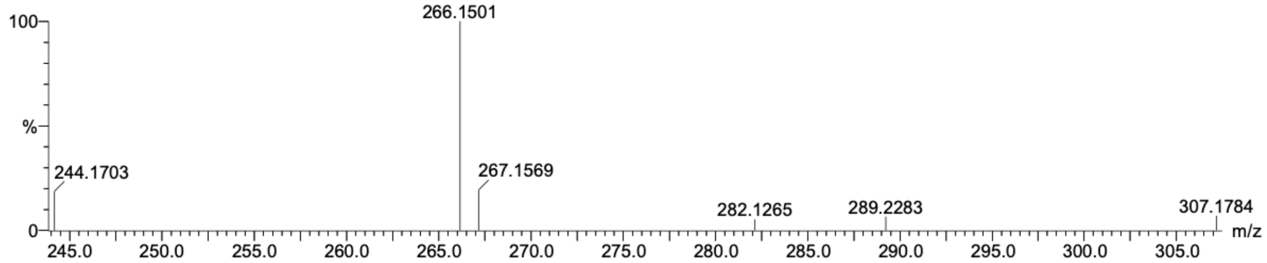

Minimum:				-1.5			
Maximum:		5.0	15.0	50.0			
Mass	Calc. Mass	mDa	PPM	DBE	i-FIT	i-FIT (Norm)	Formula
266.1501	266.1521	-2.0	-7.5	6.5	33.5	0.0	C16 H21 N O Na

Supplementary Figure 28. HRMS (ESI) spectrum of ***R*-phNA**.

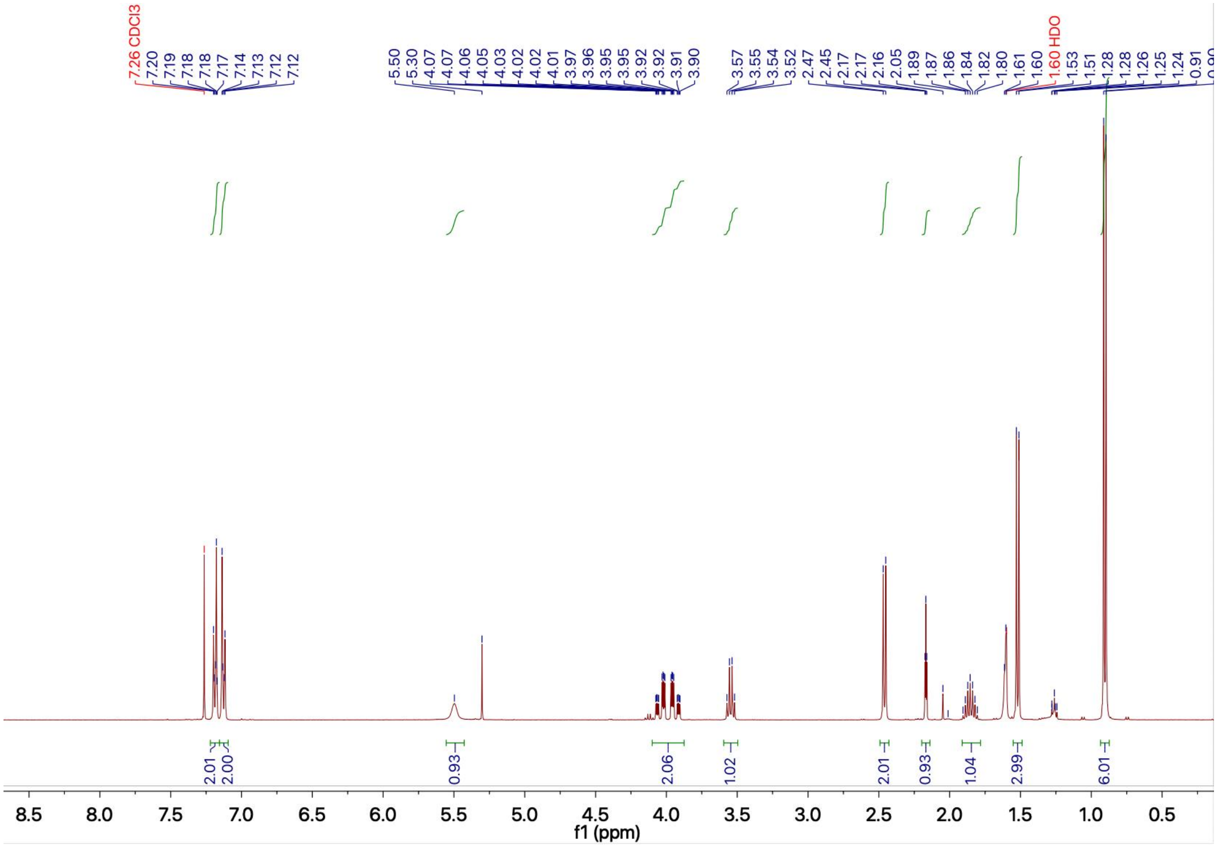

Supplementary Figure 29. ^{1}H NMR spectrum (400 MHz) of ***S*-phNA** in $CDCl_3$.
