# Supplementary material for: Photoprogrammable circularly polarized phosphorescence switching of chiral helical polyacetylene thin films
Source: Nat Commun. 2022 Dec 21;13:7841. doi: 10.1038/s41467-022-35625-3 (PMC9772410; doi:10.1038/s41467-022-35625-3)
Supplement: Supplementary file 2 — Description of Additional Supplementary Files [file 41467_2022_35625_MOESM2_ESM.pdf]

File Name: Supplementary Movie 1
Description: The photoinduced circularly polarized phosphorescence process of p($phNA_7BrNpA_3$)-PMMA under continuous UV irradiation.

File Name: Supplementary Movie 2
Description: The process of gradually illuminating the lampshade when the lamp panel is connected to the power supply.
